# Quality of Life and Events in the First Year of Life. Results from the Prospective Copenhagen Birth Cohort 1959?61

**DOI:** 10.1100/tsw.2006.14

**Published:** 2006-01-24

**Authors:** Søren Ventegodt, Trine Flensborg-Madsen, Niels Jørgen Andersen, Mohammed Morad, Joav Merrick

**Affiliations:** ^1^ Nordic School of Holistic Medicine, Teglgårdstræde 4-8, DK-1452 Copenhagen K, Denmark; ^2^ Quality of Life Research Center, Teglgårdstræde 4-8, DK-1452 Copenhagen K, Denmark; ^3^ Quality of Life Research Clinic, Teglgårdstræde 4-8, DK-1452 Copenhagen K, Denmark; ^4^ The Scandinavian Foundation for Holistic Medicine, Sandvika, Norway; ^5^ Norwegian School of Management, Sandvika, Norway; ^6^ Department of Family Medicine, Ben Gurion University of the Negev, Beer-Sheva, Israel; ^7^ Clalit Health Services, Ben Gurion University of the Negev, Beer-Sheva, Israel; ^8^ National Institute of Child Health and Human Development, Ben Gurion University of the Negev, Beer-Sheva, Israel; ^9^ Center for Multidisciplinary Research in Aging, Ben Gurion University of the Negev, Beer-Sheva, Israel; ^10^ Division of Pediatrics, Faculty of Health Sciences, Ben Gurion University of the Negev, Beer-Sheva, Israel; ^11^ Office of the Medical Director, Division for Mental Retardation, Ministry of Social Affairs, Jerusalem, Israel

**Keywords:** birth cohort, longitudinal study, maternal health, child health, human development, global quality of life, QOL, psychomotor development, institutionalization, Denmark

## Abstract

The objective of this paper was to explore the association between diverse factors occurring during the first year of a child?s life and the quality of life later as an adult. The design was a prospective cohort study based on material from the Copenhagen Birth Cohort 1959–61 with 7,222 participants and two sets of questionnaires used: one by a physician during the child's first year and one by the ?adult child? 31–33 years later. The results showed that a mother's attitude towards her pregnancy, unsuccessful abortions, and/or institutionalization left a permanent trace on the child, since these children, as adults, have a quality of life 3% below the average. Meningitis during the first year of life resulted in a quality of life 11.7% below the average, while other illnesses or accidents did not have an effect. The largest associations were found with psychomotor development, where “walking with support” showed a difference of 14.2% in overall quality of life between the fastest and slowest group. Generally, diet is not correlated with quality of life, however, we find a small, but essential, correlation between the quality of life of the adult and the early cessation of suckling (4%). Full-time institutionalization during the first year of life showed a connection with the quality of life of the adult (7.1%). It is concluded that our quality of life, health and ability as adults are primarily determined by what we ourselves choose to do with our lives as young people and as adults - and only to a marginal degree determined by factors related to our background. This suggests that we as adults have a great freedom to achieve a good life despite our experiences in the beginning of life.

## INTRODUCTION

Quality of life (QOL)[1,2,3] and its theoretical analysis are now part of the public debate[4,5,6,7, 8,9,10,11] with one of the most important questions raised about what determines the global quality of life[7]. The objective of this paper was to investigate whether the ever-present differences in quality of life of adults can be explained by differences in life circumstances in their first year of life.

Much recognition has been paid to the role of early life occurrences on adult life, examining the role that early life occurrences have on, for example, personality traits or biologic embedding[8,9]. Studies have shown that the mother's attitude towards her child is associated with the child's behavior 5 years later[10] and a series of indicators according to the mother has shown to be associated with the child's health-related quality of life at 13 years[11]. The data analyzed for this paper were taken from the Copenhagen Birth Cohort 1959–61[12], which also has been the subject of a number of prospective studies examining the effects of the first year of life on later life[13,14,15,16,17,18,19,20,21,22,23,24,25, 26,27,28,29,30].

The concept of quality of life has become an important topic in many contexts. The World Health Organization defines health as psychosocial well being and not the mere absence of disease and, therefore, in recent years, the concept of quality of life and living "a good life" has been the subject of a number of philosophical and psychological studies[4,5,6,7,8,9,10,11,31,32,33,34,35,36,37,38,39, 40,41,42]. The exploration of factors associated with quality of life is of broad importance, not only to find ways to promote people's quality of life, but also in the understanding of prevention and occurrence of illnesses.

The hypothesis for this study was that a connection exists between life experiences in the child's first year and the quality of life of the adult child 31–33 years later.

## METHODS

The connections between quality of life and the various early factors, which are presented in this study, are based on two sets of responses. The first set was from a questionnaire filled out by the physician and the mother during the first year of a child's life and the second set was completed by the grown-up "child" using the SEQOL questionnaire (Self-Evaluation of Quality of Life Questionnaire)[43,44] 31–33 years later.

The Copenhagen Birth Cohort 1959–61 began with the examination of 9,006 mothers and their 8,820 surviving children (from a total of 9,125 children) born in University Hospital of Copenhagen (Rigshospitalet) during the period 1959–1961. With the help of the CPR-register (Central Person Register), we succeeded in tracing 7,222 of the original children from the cohort. To ensure that the people drawn from the register were actually the same persons in the original cohort, names and dates of birth were checked, as were the names and dates of birth for their mothers. When we repeatedly compared these data, we found a match between the persons of around 98–99% and, therefore, estimate that the accurateness of the match of "the responses 1959–61" against "the responses 1993" was better than 98%.

The SEQOL questionnaire measures the global quality of life and is a self-administered questionnaire with items rated on five-point Likert scales. The questionnaire consists of 317 items and is based on an "integrative" theory of the quality of life. The integrative quality of life (IQOL) theory was created to organize a number of theories on the quality of life into a spectrum that spans the extremes of subjective and objective quality of life. These measures are showed below (sample questions from the questionnaire included)[43,44]:

Subjective measures:

1. Immediate, self-experienced well being ("How are you feeling?")
2. Life satisfaction ("How satisfying is your life?")
3. Happiness ("How happy are you at present?")

Existential measures:

4. Fulfillment of needs (e.g., “How well are your social needs fulfilled?”)
5. Experience of life’s temporal domains (e.g., “How do you feel when you are at home?”)
6. Experience of life’s spatial domains (“How satisfied are you with [each of five domains: self, partner, family, friends, community]”?)
7. Expression of life’s potentials [some 30 questions on extent to which they are fulfilled]

Objective measure:

8. Objective factors [some 80 questions on income, status, work, etc.]

Replies to each of the questions that constituted these measures were weighted and scored to yield computable numbers between a minimum of 0 and a maximum of 100[45,46,47,48,49,50,51,52]. These numbers were then taken as representing the quality of life of the respondent, expressed in terms of the eight different ways the quality of life has been measured by the questionnaire. Suitably weighted and scored, replies to the first part of the questionnaire constituted variables, the covariation of which the quality of life can be calculated.

Measuring quality of life has been the subject for many disagreements through time. In our research, the global quality of life — in the most broad and all-including sense — is the primary outcome measure (dependent variable). The IQOL theory made us include 113 items in the SEQOL questionnaire for the calculation of the global quality of life measurement[53], while the rest are control questions and questions giving more information about health status, sexuality, philosophy of life, lifestyle, self-perception, and social circumstances.

In this study, we had to deal with an essential problem: When the statistical connection between 113 life factors and the global quality of life was measured, we often had a contribution to the statistical covariation from the construction of the global quality of life measure. This problem turned out to be of little significance, as even the most strongly “constructed” connections did not count for more that one-fifteenth of the total connection. Still, this gave an error of up to 7% in covariation. As the large connections in our study showed, a covariation of 20% global quality of life or more, the above-mentioned error introduced by the construction of the global all-including quality of life measure was generally negligible. It is important to notice that the way our quality of life measure was constructed does not constitute a measuring problem, since we will almost always find a high correlation when n = 5–10,000 between quality of life and the many factors constituting the global quality of life or the factors related to them. However, we are not looking at the size of the correlation (the statistical significance), but the size of the statistical covariation (QOL difference in %) showing the clinical significance[52,54,55,56,57].

For validation, SEQOL was sent to 2,460 persons, 18–88 years old, randomly selected from the Danish Central Person Register (CPR-register) together with Nottingham Health profile (NHP), Sickness Impact Factor (SIP), and the test-retest reliability correlation was >0.8, Cronbach’s alpha was 0.75, correlation (r) to NHP was 0.49, to SIP 0.27 ($p < 0.05$). Adjustment for health status made the correlation to SIP stronger among the sick (r = 0.41). For SEQOL, 111 respondents were needed to detect a 3% difference in quality of life. Thus, SEQOL is valid as it showed a high level of reliability, sensitivity, and consistency[43,44].

## RESULTS (see also Table 1)

- **General factors according to the child:** The results showed that the mother’s attitude towards her pregnancy left a lasting impression on the child. Unwanted children, as adults, have a quality of life that is 3.4% below that of children who were wanted. Children born after failed abortions also showed a correlation (2.2%), as did institutionalization during the first year (7.4%). The psychosocial factors seem to dominate the biological factors. For instance, lack of oxygen during birth did not result in a reduced quality of life and neither did premature or postmature birth. No significant associations were found between quality of life and the weight of the newborn or the blood type, and only small associations were found according to gender and the length of the child (which can possible be explained because of smoking). Children who contracted meningitis in the first year of life had a quality of life 11.7% below the average, a surprisingly *large* correlation. Other illnesses hardly showed any conclusive effect. Even serious accidents, which were relatively common (for instance, falls from great heights or cranial fractures) did not show conclusive results in this study; neither did medical treatment received by the child.

- **The child's neurological state during birth:** Only very small associations were found between the quality of life of the adult and the neurological maturity and muscular development of the newborn and 1-year-old child. The speed of neuromotor development did not seem to have any influence on the quality of life of the adult and neither did legendary symptoms of brain damage such as "frog position" and the notorious "brain cry". Biological factors like "spontaneous activity during the first and the fifth day", sucking reflex, gripping reflex, plantar reflex, crawling reflex, and walking reflex showed to be quite unimportant as predictors for the quality of life of the adult.
- **Illness during the first year of life:** Here we found very small connections. Neither blood transfusions due to rhesus type, genetic malformations, anemia, heart dissonance, edema, blood spots under the skin (petechia), jaundice, or "the nurse having a really bad impression of the child on the fifth day" showed any correlation with the quality of life as a grown-up person. However, some factors were connected with quality of life to a small extent: Congenital malformations in the central nervous system, small head circumference on the first day after the birth, milk formula instead of mother's milk on the fifth day after the birth, and long stays in hospital during the first year of life. Problems with lungs, bronchi, or trachea also had an effect on the quality of life of the adult.
- **The psychomotor development of the child:** Here we examined a large number of factors that typically interest parents. The most significant association was found in relation to walking with support (14.2% difference in the overall quality of life of the adult between the best [fastest] and the worst [slowest] group). In contrast, how quickly the child can hold the head, smile, grab hold of things, sit, or crawl shows no associations with the later quality of life. To stand with and without support (5.1%/2.4%) and to walk without support (3.9%) showed small significant associations.
- **The nutrition of the child:** Generally, we found no connection with quality of life of the adult and the nutrition of the child. For instance, vitamins during the first year of life or iron treatment had no clear effect. We found no connection between quality of life and the age at which the child started to eat foods like meat, eggs, and vegetable-mash, or stopped using the bottle. Being very heavy (overweight) at 1 year of age could be an expression of inactivity during the first year of life[12] and this was correlated with a slightly lower quality of life for the adult.
- **Psychosocial factors:** In this area, we only found very small or no associations. The factors investigated were indicators such as institutionalization during the first year of life, the time when suckling was partly or completely stopped, and the number of times the child was examined (which might possibly be taken as an indication of the care and attention the child received from the parents). We also looked at the occurrence of malformations in brothers and sisters and full-time residence in a children's home during the first year of life (7.1% reduction in quality of life).
- **Vaccination:** Generally speaking, no associations were found according to vaccinations.
- **The child's development at the age of 1 year:** We find a small, but clear, association between quality of life and the ability to stand and walk at the age of 1 year (4–5% lower quality of life if the child could not stand or walk). We found small positive connections between normal reflexes at the age of 1 and quality of life.

- **Other factors at the age of 1 year:** We examined factors like physiological abnormalities as indications of neglect and feebleness to express the child's biological robustness and psychosocial vulnerability. Only the factors "very dirty skin" and "a blocked-up nose", classical expressions of neglect, showed a connection with quality of life of the adult. Deviations from the norm as regards to pulse and breathing were not connected with the later quality of life and neither were a series of other factors such as scoliosis, adduction spasms, and umbilical hernia. Valgus position of a foot and disturbances in the endocrine glands were relatively strongly connected with loss of quality of life in the adult person.

## DISCUSSION

The results obtained diminish the belief that differences in quality of life can be explained by differences in life circumstances in the first year of life. Only few of the factors examined during the first year had a significant association with the later quality of life. Among the factors that have the strongest associations with quality of life were the mother's attitude towards her pregnancy, unsuccessful abortions, institutionalization, and/or early cessation of suckling. These are all factors that notify about either negative attitude towards the child or separation between the mother and child during the first year of life. Besides that, especially meningitis and psychomotor development seemed to have a great impact on the later quality of life. It is complex to explain how the fact of having meningitis to such a high degree was associated with the later quality of life, especially when so many other complications are included. The strong association with the psychomotor development can possibly be explained according to the parents' attention for the child, since it is assumed that the psychomotor development is to a high degree reliant to the parental assistance. Psychomotor development can be explained to a high degree in terms of the level of brain stimulation and thus brain development, which is fundamental to the child's later school performance and achievements in life. The cognitive ability is perceived to be central to the individual's capacities and abilities to utilize the opportunities obtainable in life and this ability is part of the global quality of life, so the connection seems evocative.

All in all, it must be concluded, that experiences in the first year of life only in a very modest way affected the later quality of life. It is not accurate to conclude that a bad start in life is not reflected in later life, but considering the many factors that have been included in the study, its effect on the children's later quality of life was extremely modest, which is in accordance with other studies we have conducted. Examining close to 10,000 Danes, we showed that the factors most important for the global quality of life were good relations, with the close as well as the distant world, and the person's overall view of life[46,47,48,49,50,51,52,53,54,55]. What one possesses in objective terms — money, status, work, etc. — does not seem to be important to one's global quality of life and neither to self-assessed health. These results indicate that what is really important is not *what* you have, but *how you see, evaluate and experience what you have.* The person's level of consciousness and responsible attitude towards life and others seem far more important for the global quality of life and health, than the person's social status and material wealth. According to these findings, it is not strange that we found so few strong connections between experiences during the child's first year and their quality of life as adults. It seems to indicate that our lives as adults are determined by what we ourselves choose to do with our lives as young people and as adults, and only to a marginal degree determined by factors related to our background. This suggests that we as adults have great freedom to achieve a good life despite our experiences in the beginning of life.

**TABLE 1**
**Childs's First Year**

The connection between global quality of life (QOL) and the mother's situation during pregnancy. Only statistically ($p < 0.05$) and clinically significant factors listed. Difference in global quality of life was measured according to the IQOL theory[48] and measured with the validated SEQOL questionnaire[43,44].

| Quality of Life and ... | QOL Difference (%*) |
|---|---|
| Age when the child walked with support (before the 7th month vs. does not walk when 1 year old) | 14.2 |
| Hanging day 1; no moving | 13.4 |
| Signs of brain damage in the child (tense fontanelles vs. none known) | 12.9 |
| Malformations in the child (central nervous system) | 12.3 |
| Disease in the child's first year (meningitis) | 11.7 |
| Accidents (other than cranium trauma, poisoning, burns) vs. no accident in the child's first year | 8.9 |
| Infection in the first year (lymphatic node in armpit vs. none) | 7.9 |
| Institutionalized during the first year of the (9–10 months vs. not institutionalized) | 7.4 |
| Social status (Svalastoga's system) at the child's 1-year examination | 6.9 |
| First year of life, overview | 5.9 |
| Abnormal findings in the child at the 1-year examination (locomotive apparatus, central nervous system, respiratory system) | 5.6 |
| Does not stand at 1 year despite support | 5.1 |
| Age when child stands with support (7–8 vs. 12 months) | 4.3 |
| Traces of neglect at 1 year (child's skin very dirty) | 4.2 |
| Age when child walked without support (cannot at 1 year vs. can walk by 12th month) | 3.9 |
| Does not walk despite support at 1-year examination | 3.8 |
| Child's length at birth (45–47 vs. 50–51 cm) | 3.5 |
| Mother's attitude towards the present pregnancy (the child wanted vs. unwanted) | 3.4 |
| Start of life: overview (three domains) | 3.3 |
| The nose blocked up at the 1-year examination | 3.3 |
| Child placed in residential care after birth (vs. taken home with mother) | 3.1 |
| Breastfed vs. formula from first day | 2.9 |
| Marital status of mother at 1-year examination (married vs. unmarried) | 2.8 |
| Head circumference, first day (30–32 vs. 34–36 cm) | 2.8 |
| Start of life, overview (best/worst in three areas) | 2.7 |
| Age when child stands without support (not by 1 year vs. stands before 12th month) | 2.4 |
| Born after failed abortion | 2.2 |
| Hospitalisation in child's first year (1–2 months vs. never) | 2.2 |
| Child's height at 1 year (81–85 cm vs. less) | 2.2 |
| Gender of the child (male vs. female) | 2.1 |
| Strong physiological screaming, first day | 1.8 |
| Child's weight at 1 year (more than 12 kg vs. less) | 1.6 |
| Illness in the child's first year (lungs, bronchi/trachea) | 1.5 |
| Day care in child's first year (did not take place vs. took place) | 0.3 |
| Natural reflexes at 1 year (vs. other things) | 0.3 |
| The child's ability to sit at the 1-year examination (sit badly and bent forward vs. sit naturally) | 21.0 |

* Difference in percentage between the worst and the best (single events), or calculated with the method of weight modified linear regression (impact of all events)[52].

## CONCLUSIONS

Only few of the factors examined during the child's first year showed associations with the quality of life 31–33 years later. The mother's attitude towards her pregnancy, unsuccessful abortions, and/or institutionalization left a permanent trace on the child. Meningitis during the first year of life was connected with a quality of life 11.7% below the average, but other illnesses or accidents did not have an effect. The largest associations were found according to psychomotor development, where the factor "walking with support" showed a difference of 14.2% in overall quality of life between the fastest and slowest group. Generally, diet is not correlated with quality of life, but we found a small, but essential, correlation between the quality of life of the adult and the early cessation of suckling (4%). Full-time institutionalization during the first year of life showed a connection with the quality of life of the adult of 7.1%.

From this we conclude that occurrences in the first year of life only in a very moderate way affect the later quality of life. This is in accordance with other studies we have conducted, where your overall view of life and your attitude to what happens in your life were most important for your quality of life. Our study seemed to indicate that our lives as adults are determined by what we ourselves choose to do with our lives as young people and as adults, and only to a marginal degree determined by factors related to experiences in the beginning of life.

## ACKNOWLEDGMENTS

This study was supported by grants from The 1991 Pharmacy Foundation, as well as by supplementary grants from Goodwill-fonden, the JL-Foundation, E. Danielsen and Wife's Foundation, Emmerick Meyer's Trust, the Frimodt-Heineken Foundation, the Hede Nielsen Family Foundation, Petrus Andersens Fond, Wholesaler C.P. Frederiksens Study Trust, Else & Mogens Wedell-Wedellsborg's Foundation, and IMK Almene Fond. The research was approved by the Copenhagen Scientific Ethical Committee under number (KF) V.100.2123/91.

## BIOSKETCHES

**Søren Ventegodt, MD,** is the director of the Nordic School of Holistic Health and Quality of Life Research Center in Copenhagen, Denmark. He is also responsible for a Clinical Research Clinic for Holistic Medicine in Copenhagen and is a popular speaker throughout Scandinavia. He has published numerous scientific or popular articles and a number of books on holistic medicine, quality of life, and quality of working life. His most important scientific contributions are the comprehensive SEQOL questionnaire, the very short QOL5 questionnaire, the integrated QOL theory, the holistic process theory, the life mission theory, and the ongoing Danish Quality of Life Research Survey, 1991–94 in cooperation with the University Hospital of Copenhagen and the late professor of pediatrics, Bengt Zachau-Christiansen, MD, PhD. E-mail: ventegodt@livskvalitet.org. Website: www.livskvalitet.org

**Trine Flensborg-Madsen**, **BSc**, is a research assistant at the Quality of Life Research Center in Copenhagen. She is currently finishing her Master of Public Health (MPH) at the Department of Public Health, University of Copenhagen, Denmark. E-mail: tfm@livskvalitet.org

**Niels Jørgen Andersen, MSc,** professor, Department of Innovation and Economic Organization, Norwegian School of Management. This department conducts research and provides teaching in central topics related to innovation, business development, management of global companies, business history, and economic organization. Research activities within the Department are related to four core subjects within the discipline: business history, cooperative organizations, business development and entrepreneurship, and finally studies of industries with a special focus on the electricity industry. He is also the dynamic chairman of the nonprofit organization Stiftelsen Holistisk Medisin Scandinavia, which aims to support the scientific development, research, and documentation of complementary and holistic medicine in Scandinavia. E-mail: niels.j.andersen@bi.no. Website: www.bi.no/users/fgl93013/

**Mohammed Morad, MD**, is specialist in family medicine, lecturer in family medicine affiliated with the Department of Family Medicine, Division of Community Health, National Institute of Child Health and Human Development and Center for Multidisciplinary Research in Aging, Faculty of Health Sciences, Ben Gurion University of the Negev and the medical director of a large area clinic in the city of Beer-Sheva operated by the Clalit Health Services. Dr. Morad has numerous publications on Bedouin health, health aspects, spiritual health, and aging in persons with intellectual disability and is a presenter at national and international conferences on topics such as health policy and services for the disadvantaged. E-mail: morad@bgu.ac.il

**Joav Merrick, MD, DMSc,** is professor of child health and human development affiliated with the Center for Multidisciplinary Research in Aging, Zusman Child Development Center, Division of Pediatrics and Community Health at the Ben Gurion University, Beer-Sheva, Israel; the medical director of the Division for Mental Retardation, Ministry of Social Affairs, Jerusalem; and the founder and director of the National Institute of Child Health and Human Development. Dr. Merrick has numerous publications in the field of child health and human development, rehabilitation, intellectual disability, disability, health, welfare, abuse, advocacy, quality of life, and prevention and he received the Peter Sabroe Child Award for outstanding work on behalf of Danish Children in 1985 and the International LEGO-Prize ("The Children's Nobel Prize") for an extraordinary contribution towards improvement in child welfare and well being in 1987. E-mail: jmerrick@internet-zahav.net. Website: www.nichd-israel.com
